# A predicted protein interactome identifies conserved global networks and disease resistance subnetworks in maize

**DOI:** 10.3389/fgene.2015.00201

**Published:** 2015-06-04

**Authors:** Bryan Musungu, Deepak Bhatnagar, Robert L. Brown, Ahmad M. Fakhoury, Matt Geisler

**Affiliations:** ^1^Department of Plant Biology, Southern Illinois UniversityCarbondale, IL, USA; ^2^Food and Feed Safety Research, Southern Regional Research Center, United States Department of Agriculture, Agricultural Research ServiceNew Orleans, LA, USA; ^3^Department of Plant Soil and Agriculture Systems, Southern Illinois UniversityCarbondale, IL, USA

**Keywords:** Zea mays, interactome, network, protein-protein interactions, disease resistance, tool

## Abstract

Interactomes are genome-wide roadmaps of protein-protein interactions. They have been produced for humans, yeast, the fruit fly, and *Arabidopsis thaliana* and have become invaluable tools for generating and testing hypotheses. A predicted interactome for *Zea mays* (PiZeaM) is presented here as an aid to the research community for this valuable crop species. PiZeaM was built using a proven method of interologs (interacting orthologs) that were identified using both one-to-one and many-to-many orthology between genomes of maize and reference species. Where both maize orthologs occurred for an experimentally determined interaction in the reference species, we predicted a likely interaction in maize. A total of 49,026 unique interactions for 6004 maize proteins were predicted. These interactions are enriched for processes that are evolutionarily conserved, but include many otherwise poorly annotated proteins in maize. The predicted maize interactions were further analyzed by comparing annotation of interacting proteins, including different layers of ontology. A map of pairwise gene co-expression was also generated and compared to predicted interactions. Two global subnetworks were constructed for highly conserved interactions. These subnetworks showed clear clustering of proteins by function. Another subnetwork was created for disease response using a bait and prey strategy to capture interacting partners for proteins that respond to other organisms. Closer examination of this subnetwork revealed the connectivity between biotic and abiotic hormone stress pathways. We believe PiZeaM will provide a useful tool for the prediction of protein function and analysis of pathways for *Z. mays* researchers and is presented in this paper as a reference tool for the exploration of protein interactions in maize.

## Introduction

Understanding the biological interactions within an organism is vital for the comprehension of its functions. The interactome provides a large scale mapping of protein-protein interactions (PPIs). Interactomes of model organisms such as *Arabidopsis thaliana* and *Saccharomyces cerevisiae* were built using high throughput experimental methodologies (Consortium, 2011). However, predicted interactomes in species of agronomic importance, like *Citrus sinensis*, *Oryza sativa*, and *Glycine max*, have provided insight into disease resistance. There was no plant interactome until the *Arabidopsis* predicted interactome was released in 2007 (Geisler-Lee et al., 2007). It was based on orthologs (genes separated by speciation) of *S. cerevisiae* (Yu et al., 2008), *Drosophila melanogaster* (Giot et al., 2003), *Caenorhabditis elegans* (Li et al., 2004), and *Homo sapiens* (Rual et al., 2005). This predicted plant interactome successfully provided hypotheses for testing interactions, including those involving membrane proteins, which are otherwise difficult to elucidate using forward and reverse genetic approaches (Lalonde et al., 2010; Nejad et al., 2012). Although experiment-based interactomes for *A. thaliana* are now being made (Consortium, 2011; Chen et al., 2012), the predicted interactome still makes many useful predictions for interactions not yet found in the growing experimental dataset. For instance, studies by Guo et al. (2009) which tackled the complexity of germination and the involvement of plant hormone pathways, found interacting partners of Rack1 (receptor for activated kinases1) from a candidate list of 88 partners using a predicted interactome. Plant predicted interactomes have also aided in determining proteins involved in resistance to the destructive bacterial pathogen Huanglongbing in citrus (Martinelli et al., 2012, 2013), as well as to the soybean cyst nematode (SCN) in soybean (Lightfoot, 2014). Moreover, the human interactome was used to link the differential expression of genes with protein interactions in the analysis of cancer tissues, allowing researchers to analyze the connectivity between known and novel targets (Wachi et al., 2005). Thus, interactomes allow for hypotheses to be generated with *a posteriori* and *a priori* knowledge of a biological system.

The underlying principle for a predicted interactome is that evolutionarily conserved proteins tend to have conserved interactions when the proteins retain orthologous functions. Software programs such as Inparanoid (Ostlund et al., 2010), OrthoMCL (Li et al., 2003), and MSOAR (Geer et al., 2010), along with many others, have been developed in order to discover all orthologs and outparalogs (duplications prior to divergence of species) between two or more genomes, and to separate these from inparalogs (duplication within a lineage). PPIs can thus be predicted across an entire genome by high throughput computational methods using whole genome ortholog prediction (Geisler-Lee et al., 2007; Schuette et al., 2015). These methods have been successfully used to predict interactomes for *A. thaliana*, *G. max*, *Coffea robusta*, *H. sapiens*, *C. sinensis*, *D. melanogaster*, *O. sativa* (rice), and *P. patens* (a moss) (Giot et al., 2003; Li et al., 2004; Brown and Jurisica, 2005; Rual et al., 2005; Guan et al., 2008; Consortium, 2011; Geisler and Fitzek, 2011; Gu et al., 2011; Ho et al., 2012; Ding et al., 2014; Lightfoot, 2014). Moreover, physically interacting proteins tend to be encoded by genes co-expressed in response to different stimuli in many species (Giot et al., 2003; Bhardwaj and Lu, 2005; Rual et al., 2005). Expression data, such as microarray and RNA-Seq, can thus be used as an additional layer of support for PPIs predicted through orthology.

An interactome can be visualized as a field of circles (nodes) that represent proteins and connections (edges) between nodes representing PPIs. Each node can be rated based on the number of connections, referred to as the connectivity or degree of that node. Protein interactomes typically contain a few highly connected hubs (proteins with >10 partners) and numerous smaller hubs (proteins with 3–10 partners), pipes (2 partners) and free ends (1 partner only). This distribution is an inverse power relationship between node frequency and connectivity, and is similar to that of other small-world network structures such as social networks and electrical power grids (Watts and Strogatz, 1998; De Silva et al., 2006; Gu et al., 2011). The small-world topology is a compromise between efficiency and robustness. Having fewer interacting partners involved in a pathway results in increased efficiency in terms of how fast a product or outcome can be processed. The highly connected hubs represent proteins that are conserved through different organisms and are under less selection pressure for mutations (Batada et al., 2006; Zotenko et al., 2008; Ning et al., 2010). For example, heat shock proteins (i.e. Hsp70) are some of the highly connected hubs in most interactome networks (Gopinath et al., 2014). These proteins are vital for helping other proteins to fold properly, assemble, and translocate. They have also been implicated in reactions to abiotic and biotic stress (Wang et al., 2004). A robust Hsp70 system includes redundant pathways, auto-regulation, and feedback for increased stability.

*Zea mays* (maize) is one of the three major global crops (FAOstat, 2009; Ranum et al., 2014). Although its genome was sequenced (Schnable et al., 2009) a few years ago, it still contains many poorly annotated genes with provisional functional annotation based on sequence or domain homology. In addition, proteins with a known biological role in a species are often found to have, in another species, even more biological roles that were previously unknown or overlooked. A systematic approach to the analysis of the connectivity between known and novel proteins will help identify these respective biological roles, and will add to existing *Z. mays* gene annotation. PiZeaM is thus a useful tool to identify networks of connections among proteins in many key *Z. mays* processes. The recent increase in the amount of genome and interactome data for model organisms has made it possible to systematically predict PPIs in *Z. mays* from experimentally determined reference PPIs maps.

In addition to gene discovery and functional annotation, the ultimate goal in systems biology is to gain an understanding of the networks of molecular processes in an organism. These systems biology efforts at network analysis have begun in model organisms such as *A. thaliana* (Consortium, 2011), and *Synechocystis sp. PCC6803* (Kim et al., 2008). However, where these efforts are urgently needed is in the major cereal crops. *Z. mays* is one of the three major food crops. Moreover, over the last decade, there has been an increase in the use of *Z. mays* to produce the biofuel ethanol as a substitute to gasoline. In fact, it has been estimated by the Renewable Fuels Association that 26% of the *Z. mays* grown in the US in 2012 was used for the production of ethanol (RFA, 2012). Increasing the yield in *Z. mays* through better management practices and improved hybrid genetics is a goal actively sought by the researchers around the world.

In this work we present PiZeaM—a predicted *Z. mays* interactome—to help functional annotation of gene encoding proteins, to understand their connectivity and to aid systems biology approaches. This interactome can expand knowledge of protein functions by analysis of interacting proteins' connectivity and by providing maps of interconnected pathways. We demonstrated the accuracy of the published prediction method by comparing the predicted *A. thaliana* interactome in 2007 with the experimentally determined interactomes published in 2008–2014. We believe PiZeaM will provide a useful tool for the prediction of protein function and analysis of pathways for *Z. mays* researchers.

## Materials and methods

### Choice of prediction method

The interolog method was chosen to predict protein interactions in *Z. mays* due to its success in other plant species (Gu et al., 2011; Ho et al., 2012), including *O. sativa*, another member of the grass family. Bioinformatic algorithms and programs, and corresponding parameters and weights used to produce this interactome were the same as those used in *O. sativa* (Ho et al., 2012).

### Sources of protein sequences

Amino acid sequences from *Z. mays* (ZmB73 4a.53 sequence) were retrieved from the GRAMENE database (http://www.gramene.org; Chandler and Brendel, 2002; Youens-Clark et al., 2011). The accession numbers were later mapped onto a more recent version ZmB73 5a to gain access to updated annotation tools in Phytozome (phytozome.jgi.doe.gov). Those of the 13 reference species—nine eukaryotes *Oryza sativa*, *Arabidopsis thaliana*, *Homo sapiens*, *Mus musculus*, *Rattus norvegicus*, *Drosophila melanogaster*, *Caenorhabditis elegans*, *Saccharomyces cerevisiae*, *Schizosaccharomyces pombe*, and four prokaryotes *Escherichia coli*, *Bacillus subtilis*, *Helicobacter pylori*, *Campylobacter jejuni*, and *Synechococcus*—were retrieved from ENSEMBL www.ensembl.org/index.html (access date November 2011), (Flicek et al., 2012) and NCBI http://www.ncbi.nlm.nih.gov, (Geer et al., 2010).

### Prediction of orthologs

The amino acid sequences were then processed with the Linux based program Inparanoid 3.0 (Ostlund et al., 2010). BLOSUM (BLOcks SUbstitution Matrix) 80 was used for three plant species, *A. thaliana*, *O. sativa*, and *Z. mays*; BLOSUM 62 for five animal species *C. elegans*, *D. melanogaster*, *H. sapiens*, *M. musculus*, *R. norvegicus*, and two fungal species *S. pombe*, and *S. cerevisiae*; and BLOSUM 45 for the four prokaryotes *B. subtilis*, *C. jejuni*, *E. coli*, and *Synechococcus*. One ortholog pair with a bootstrap score of 100% was chosen from each ortholog cluster for the one-to-one interactome. A second interactome was built with all pairwise combination of proteins in each ortholog cluster referred to as the many-to-many interactome. The latter allowed all potential inparalogs in both *Z. mays* and reference species to substitute for one another, while outparalogs are in separate gene clusters. Outparalogs will retain interactions within their cluster but not to other outparalogs. Gene orthology, unlike homology by blast score or “% similarity,” is considered to be a better predictor of the conservation of function, and thus conservation of protein interactions (Koonin, 2005; Ostlund et al., 2010). The number of orthologs found between *Z. mays* and the reference organisms was dependent on the evolutionary distance, and often more distantly related organisms (like bacteria and animals) had fewer orthologs with *Z. mays*.

### Predicting maize interactions from conserved orthologs

PiZeaM was built from a comprehensive analysis of physical interactions between proteins of *Z. mays* that were predicted based on experimentally determined interactions from five major interactome databases [BioGRID (verision 3.1.84; www.thebiogrid.org) (Stark et al., 2006); DIP (November 2011 release) (Salwinski et al., 2004); IntAct (downloaded November 5, 2011; http://www.ebi.ac.uk/intact) (Aranda et al., 2010); and MINT (downloaded November 5, 2011; http://mint.bio.uniroma2.it/mint) (Chatr-Aryamontri et al., 2007)]. The interactome thus consisted of a large set of data, listing pairs of interacting proteins (see Supplemental Table 1). The entire interactome can be visualized graphically using the Cytoscape software packages (Shannon et al., 2003; Cline et al., 2007) in which proteins are indicated by 2 dimensional shapes (circles) and interactions are indicated by connecting lines. Heterologous interactions were ordered (larger ID as protein A: smaller ID as protein B) as all interactions were considered bidirectional. Duplicates were then removed from the master database to produce a unique interaction table where each interaction is represented once.

### Calculation of the confidence value (CV) of experimental support

To determine the confidence value (CV), the following formula was used (Geisler-Lee et al., 2007):

CV=N×S×E

**Table:** 

N:	the number of times that an interaction appeared in PiZeaM.
E:	the number of times an experiment appeared with an interaction in the mined interactomes.
S:	the number of times an interaction appeared in the reference species.

A scale was then used to rank the CV of the interactions that comprise the interactome. This last factor (E) was an important consideration as different methods for determining protein interactions will not always give converging results rates (for example yeast-2-hybrid false positives are not likely to be the same as those that occur using *in vitro* co-precipitation or peptide arrays)(Yu et al., 2008). Protein pairs that had a *CV* = 1 were considered to have low confidence interactions, a *CV* = 2–10 was considered to indicate a medium confidence interaction, and a *CV* > 10 was considered to represent a high confidence interaction. Interactions with the highest CVs were more likely to be conserved across species.

### Mapping of maize orthologs to gene ontology

Gene ontology (GO) information from *Z. mays* was downloaded from ENSEMBL (www.ensembl.org/index.html) (Flicek et al., 2012), Phytozome Version 10.1 (http://phytozome.jgi.doe.gov/pz/portal.html), and GRAMENE Release 45 (http://www.gramene.org). These GO terms described the biological function (process), subcellular localization (compartment) and molecular function of each *Z. mays* protein, based on experimental evidence or on prediction based on protein domain composition, signal sequences and global homology (Carbon et al., 2009). Then, a custom ontology file and a reference annotation were developed for updated annotation. GO-term enrichment analysis of the interactions within PiZeaM was conducted by comparing proteins within the interactome (6004 proteins) with the whole genome of *Z. mays*. This comparison established which biological functions were captured by the aforementioned interolog method and determined which types of proteins were conserved as interacting ortholog pairs. A second GO comparison was made of 1st neighbors (prey) of proteins with the GO term “response to other organism” (GO: 0051707) (bait). The Cytoscape Bingo plugin was then used to look at enrichment in the predicted interactome contrasted with the whole annotation (Maere et al., 2005). The GO terms were then sorted by their enrichment or depletion *P*-value, and for terms reflecting molecular function, biological process, and cellular component (Supplemental Table 3).

### Co-expression analysis

For expression data analysis and determination of co-expression between various genes, microarray data was downloaded from the Gene Expression Omnibus (Edgar et al., 2002) from 68 biotic and abiotic stress data sets generated using the same Affymetrix *Z. mays* Gene chip (see Supplemental Table 2). To generate a co-expression matrix, the change in levels of mRNA was normalized using global intensity normalization and computed as an M-value (Log base 2 of the ratio of stimulus over control).

$$\left[M-value=log_{2}\left(\frac{treatment}{control}\right)\right]$$

Pearson correlation was calculated for each of the data sets using the R statistical language (Edgar et al., 2002; Stuart et al., 2003; Bhardwaj and Lu, 2005). The correlation function was used to determine the Pearson correlation between random proteins and true interologs. Pearson correlation coefficient (r):

$$\text{r}=\frac{\text{n}(\sum \text{xy})-(\sum \text{x})(\sum \text{y})}{\sqrt{\left[\text{n}\sum {\text{x}}^{2}-(\sum {\text{x}}^{2})][\text{n}\sum {\text{y}}^{2}-{\left(\sum \text{y}\right)}^{2}\right]}}$$

**Table:** 

N:	the number of expression samples.
X:	the expression level of gene X in ith sample.
Y:	the expression level of gene Y in the ith sample.

The value of r is between −1 and 1 (i.e. −1 < r < 1). A Pearson correlation coefficient (r) was generated for each pair of interacting proteins in the interactome. Interacting proteins that had a high *r*-value were considered to be more likely to be coordinately co-expressed (Narayanan et al., 2010).

### Visualization of PiZeaM

One objective of this study was to make PiZeaM readily available for other researchers to use. To visualize PiZeaM, Cytoscape 3 (Cline et al., 2007) can be used by importing the provided Excel files (Supplemental Table 1), or by opening the compiled Cytoscape file (Supplemental File 1). The information can be easily stored and manipulated in MySQL (a database language) and Microsoft Excel to address different research needs. To visualize a protein of choice from a pathway, only requires retrieving protein IDs for the *Z. mays* proteins of choice from Ensemble. One limitation of the interactome is a possible under-representation of interactions exclusive to *Z. mays*, since a substantial amount of the data used in this study to build the interactome was derived from interactions reported in non-grasses. This limitation can be alleviated in the future by incorporating more data from future research involving grasses and cereals, once these experimental datasets are built and shared with the public.

## Results

### Overview of PiZeaM

Using one-to-one interolog as a method of predicting interactions, a total of 34,107 unique interactions were found for 4843 *Z. mays* proteins from the 110,185 *Z. mays* proteins tested (Supplemental Table 1). A summary of the relative contribution of each reference species to the predicted interactions is shown in (Table 1). PiZeaM represented less than 5% of the *Z. mays* proteome due to the exclusion of paralogous and duplicated genes, which constitute a relatively large proportion of the *Z. mays* genome. When duplicated genes are included in the prediction of the interactome, using a many-to-many ortholog matching method that allows the inclusion of paralogs, 1161 *Z. mays* proteins that were only in the many-to-many set, as well as 14,919 unique interologs were added to the unique interactome. This resulted in a combined 49,026 unique interactions comprising 6004 *Z. mays* proteins (Table 2). A premade cytoscape formatted graphical visualization of PiZeaM for this combined set of proteins is included in Supplemental File 1).

**Table 1:** **Reference organism data used for maize interolog prediction**.

**Organism**	**Total Proteins**	1 to 1 interologs^*^	M to M interologs^*^	**M to M fold change**	**Total interactions**
*Arabidopsis thaliana*	33602	4289	7331	1.709256237	11620
*Saccharomyces cerevisiae*	7126	42250	51873	1.227763314	94123
*Drosophila melanogaster*	15246	1508	2232	1.480106101	3740
*Rattus norvegicus*	29516	22	78	3.545454545	100
*Mus musculus*	37991	105	269	2.561904762	374
*Schizosaccharomyces pombe*	7031	2766	3121	1.128344179	5887
*Homo sapiens*	57945	2735	5005	1.829981718	7740
*Bacillus subtilis*	4371	32	32	1	64
*Caenorhabditis elegans*	20447	1	1	1	2
*Campylobacter jejuni*	3673	60	923	15.38333333	983
*Synechococystis*	3264	161	501	3.111801242	662
*Oryza Sativa*	58058	220	248	1.127272727	468
*Escherichia coli K12*	4618	2496	2444	0.979166667	4940
Total					130703

* *Only orthologs used for interologs are listed*.

**Table 2:** **Predicted maize interactions**.

**Orthology**	**Proteins**	**Interactions**
Combined Total (One to One/Many to Many)	6004	130703
One to One Total	4843	56645
Many to Many Total	5673	74058
Combined Unique (One to One/Many to Many)	6004	49026
One to One Unique	331	4737
Many to Many Unique	1161	14919
Overlap between Unique (One to One/Many to Many)	4512	29370

### Confidence value, conservation and connectivity in predicted *Z. mays* interactions

The initial analysis of PiZeaM was performed to determine accuracy and to determine if the network of predicted protein interactions in a major plant crop species such as *Z. mays* had the same structure as those described for model organisms such as *A. thaliana* and *S. cerevisiae*. This analysis helped in establishing and differentiating the value of each predicted interaction and protein in the network, and allowed for determining the weights within the sub-networks.

Confidence values (CVs) for each interaction in PiZeaM are listed in Supplemental Table 1 and added to the network visualization in Supplemental File 1 as an edge feature. PiZeaM had 1079, 38,851, and 9096 low, medium and high confidence interactions, respectively. Thus most interactions were at least medium confidence with more than one supporting line of evidence.

These levels of confidence allow users to select specific levels of false discovery when the data is used to build networks or to develop hypotheses. The most confident interactions were self-interactions for AAA ATPases, Topoisomerases and DNA-Repair (Supplemental Table 1). On the other hand, the most confident hetero-interactions were with proteins involved in core molecular processes, such as elements of the DNA repair machinery, the basal promoter complex, the proteosome, and proteins associated with the regulation of the cell cycle (Table 3). DNA repair machinery is conserved throughout eukaryotes (Liu et al., 1999; Ohbayashi et al., 1999) and was recovered in PiZeaM (Supplemental File 1). High CV correlates with conserved strong interactions that are detected by multiple methodologies in different species.

**Table 3:** The most confident hetero-interactions in the predicted maize interactome^*^.

Protein A^**^	Protein B^**^	**Protein A description**	**Protein B description**	**CV**
008327_P01	061287_P01	Cell division control protein 2 homolog	Cyclin superfamily protein, putative	12960
056075_P01	110212_P01	DNA mismatch repair protein MSH2	MUS2 protein	10752
AC149818.2_FGP004	008327_P01	Cyclin-dependent kinases regulatory subunit	Cell division control protein 2 homolog	9450
045314_P01	064732_P01	Guanine nucleotide-binding protein subunit beta	G protein alpha subunit 1	6720
008327_P01	017081_P01	Cell division control protein 2 homolog	Uncharacterized protein	6120
033626_P02	061745_P01	26S proteasome non-ATPase regulatory subunit 14	Uncharacterized protein	5880
093050_P01	174757_P01	Eukaryotic translation initiation factor 3 subunit A	Uncharacterized protein	5772
040152_P02	088162_P01	Unknown	Putative uncharacterized protein	5760
047774_P01	105409_P02	Uncharacterized protein	Unknown	5616
028313_P03	452026_P01	Putative translation elongation/initiation factor	Eukaryotic peptide chain release factor subunit 1-1	5124
071518_P01	085970_P01	Spc97/Spc98 family of spindle pole body (SBP) component	Tubulin gamma-2 chain	5040
046021_P01	177974_P02	Histone acetyltransferase of the GNAT family 1	Uncharacterized protein	4320
043484_P01	148924_P02	Histone-lysine N-methyltransferase EZ3	Polycomb group protein FIE2	3696
161418_P02	168096_P01	TATA-box-binding protein 2	DNA binding	3640
021069_P01	066101_P02	Minichromosome maintenance protein	Unknown	3584
133952_P01	356935_P01	MUTL-homolog 1	Uncharacterized protein	3432
AC234199.1_FGP003	048067_P01	Unknown	Uncharacterized protein	3360
152328_P01	556845_P01	Actin-1	Unknown	3315
054225_P01	402295_P01	DNA-directed RNA polymerase	Unknown	3312
042371_P01	100872_P01	Elongator protein 2	Uncharacterized protein	3024

* Unique one-to-one interactome interactions with the highest confidence values.

** *GRMZM2G prefix for identifiers removed for space*.

“*Connectivity*” of PPIs determines the number of interacting partners for a given protein. Proteins with many interacting partners, referred to as having “*high connectivity*,” are of biological interest as they may represent the “circuit hubs” central to signaling and information processing in the organism. The distribution of the connectivity of elements in most information systems has implications on features such as robustness and efficiency of the system (Alon, 2007). In the PiZeaM, the highly connected proteins were ubiquitous partners and co-factors such as chaperones, scaffolding proteins, and protein involved in degradation pathways (see Table 4).

**Table 4:** **The most connected maize proteins**.

**Protein ID**	**Annotation**	Connectivity^*^
GRMZM2G118637_P01	Putative ubiquitin family protein	797
GRMZM2G012280_P01	Uncharacterized protein	523
GRMZM2G033626_P02	26S proteasome non-ATPase regulatory subunit 14	496
GRMZM2G012631_P01	HSP protein	432
GRMZM2G327635_P01	Ubiquitin carboxyl-terminal hydrolase	399
GRMZM2G147671_P01	26S proteasome non-ATPase regulatory subunit 4	348
GRMZM2G416120_P01	Chaperonin CPN60-2l	334
GRMZM2G155384_P02	Uncharacterized protein	324
GRMZM2G075719_P02	Uncharacterized protein	308
GRMZM2G046021_P01	histone acetyltransferase of the GNAT family 1	281
GRMZM2G152328_P01	Actin-1	274
GRMZM2G008410_P01	Transcribed sequence 1087 protein	250
GRMZM2G038964_P02	Uncharacterized protein	248
GRMZM2G153815_P01	Heat shock 70 kDa protein	248
GRMZM2G171080_P01	Uncharacterized protein	232
GRMZM2G008327_P01	Cell division control protein 2 homolog	230
GRMZM2G027282_P01	Uncharacterized protein	230
GRMZM2G303752_P01	ATP-dependent rRNA helicase spb4	215
GRMZM2G014676_P01	Prefoldin 5	199
GRMZM2G107540_P01	Core histone H2A/H2B/H3/H4 domain	195

* *Number of predicted interacting protein partners for the unique interactome*.

Highly connected hubs are reported to represent the most evolutionarily conserved proteins and to form the backbone of core processes (Evlampiev and Isambert, 2008). Proteins in *Z. mays* with a large number of interacting partners were also found to be highly conserved (Table 4). The protein with the highest connectivity was Ubiquitin 2 (GRMZM2G118637_P01), with 797 different predicted protein partners. This protein is orthologous to ubiquitin in *S. cerevisiae*, involved in targeted protein degradation. In *A. thaliana*, ubiquitination has been shown to play a role in abiotic stresses, biotic stresses, and in other cellular processes, including auxin based growth stimulation (Dong et al., 2006). Some of the other highly interconnected conserved proteins are chaperonins, heat shock proteins, and members of large protein complexes such as the ribosome and the proteosome. Specific conserved interactions were similar to those found in *A. thaliana*, including interactions between histones, proteosome components, MutS type DNA repair proteins, and tubulin binding to the spindle pole body Spc97/Spc98. Thus, in *Z. mays*, despite the large changes in genome structure and increased number of genes compared to the reference species used, the conserved interactions remain intact, and the hypothesis that highly connected hubs have deeply conserved interactions across species, genera and even kingdoms (Bork et al., 2004; Consortium, 2011) is also evident in domesticated crop species like *Z. mays*.

Phosphorylations of Serine-threonine/tyrosine-protein kinases and their connected transcription factors are instrumental in signaling pathways involved in modulating responses to abiotic and biotic stresses, cell growth, and development (Rudrabhatla et al., 2006). These transcription factors and signaling proteins are underrepresented in the *Z. mays* interactome, although family members are found in all used reference organisms. For example the protein serine/threonine kinase activity (GO:0004674) is found 1135 times in the whole maize genome while only 181 times in the interactome (it was expected 299.3 times based on size of population sample), thus is 1.65-fold depleted. This indicates a lack of conservation of their interactions despite their key importance, and likely represents lineage specific rewiring of regulatory networks. Changes in differential gene regulation are thought to be the earliest step in evolutionary divergence, followed only later by changes in gene function (Evlampiev and Isambert, 2008). Some regulatory processes (ubiquitination, methylation, chromatin remodeling) are highly conserved, and more importantly, maintain their conserved interacting partners and complexes throughout eukaryotic evolution (Hershko and Ciechanover, 1998; Kaiser and Huang, 2005; Soltes et al., 2011). These may represent only the core mechanisms for regulation, while the specificity of gene and protein targets in other pathways such as map kinases may diverge or rewire over the course of evolution (Mosca et al., 2012).

### Topology of PiZeaM

**Figure 1:**
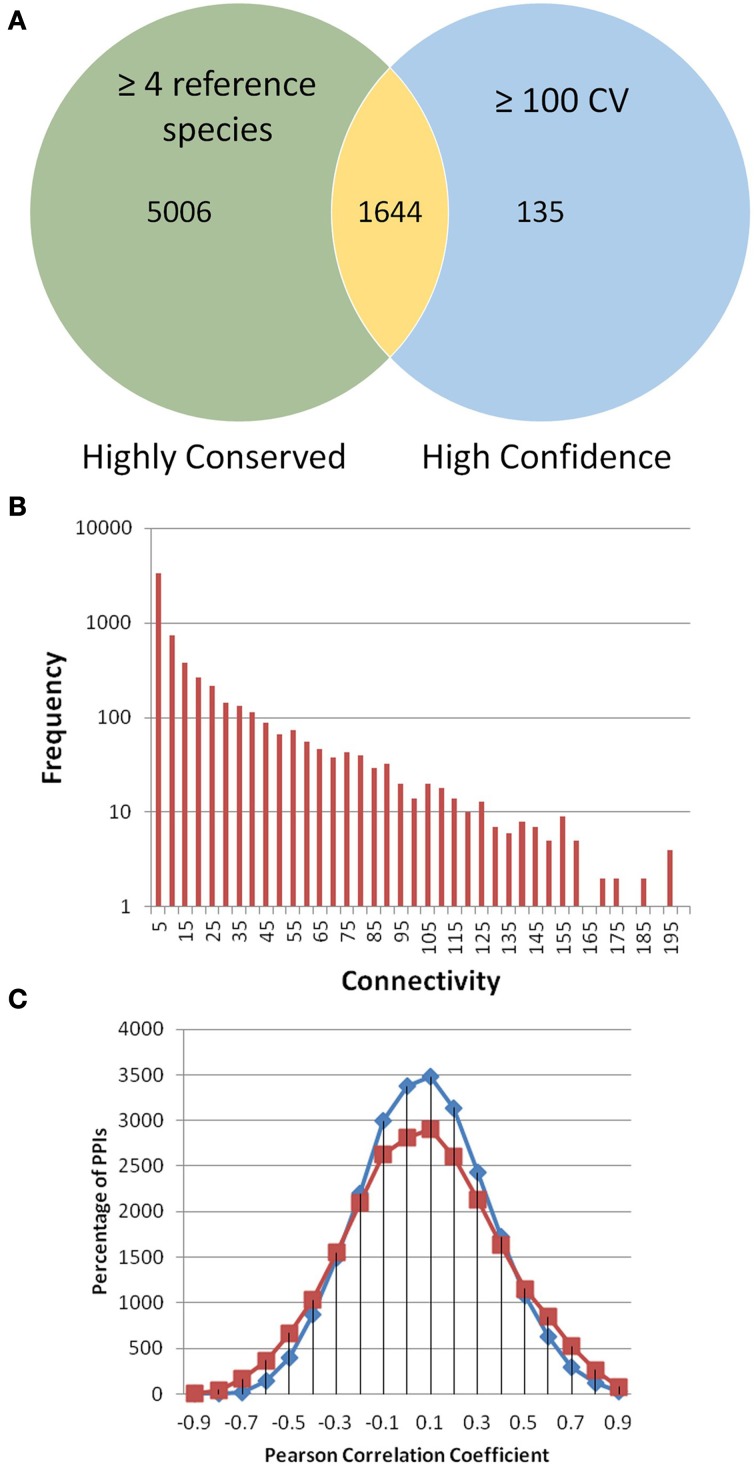
**PiZeaM toplogy and co-expression**. All unique interactions in PiZeaM (merged one-to-one and many-to-many orthology) were analyzed. **(A)** Highly conserved interactions with interologs from four or more different reference species interactomes were compared to interactions with greater than or equal to a confidence value (CV) 100. The minimum CV of an interaction with 4 reference species is 16. Larger CVs indicate additional support from different experimental methodologies or replication. **(B)** Frequency distribution of proteins with increasing numbers of unique interacting partners (including self-interaction) is plotted on a log-linear graph. **(C)** Frequency distribution of gene transcript expression (Pearson correlation of M-values from 68 microarrays) for interacting protein pairs (red) and random pairs of proteins from those found in the interactome (blue). Random interactions are statistically significantly different from nonrandom interactions (*P*-value of 9.53644 × 10^−39^ in a student *t*-test), although the average correlation coefficient of both distributions is similar, observed correlations were more widely spread at both high positive and negative ends.

Topology looks at patterns in networks and compares network properties. The frequency distribution for node connectivity was calculated for PiZeaM (Figure 1B). The majority of proteins were intermediate sized hubs with 10–100 interacting protein partners. The unique interactome had an average connectivity of 15.86 neighbors per node, similar to *D. melanogaster* and *O. sativa* (Giot et al., 2003; Stark et al., 2006; Gu et al., 2011). The distribution of nodes by connectivity (Supplemental Table 1) follows an inverse power relationship between node frequency and connectivity, which is typical of “small world” topology networks, and is frequently seen in biological and social networks, and in transportation hubs (Watts and Strogatz, 1998; Alon, 2007).

The shortest path length within a network is the path with the fewest number of intervening nodes between two given nodes. When all protein pairs in PiZeaM were evaluated there was an enriched frequency of path lengths between 2 and 4 with a characteristic path length of 3.942 intervening nodes (Net.Stat in Supplemental File 1). Only a small number of protein pairs had a path length greater than 6. These pathways suggest that a majority of interactions can be characterized as short path interactions (shown in Supplemental Figure 1). This is similar to the 2.6 node characteristic path length of *S. cerevisiae* (Gursoy et al., 2008). The *A. thaliana* interactome has a characteristic path length of 3.4 (Chen et al., 2012) and the human interactome has a characteristic path length that varies among databases between 1 and 3 (Taylor et al., 2009). The overall topology of the PiZeaM resembles that of experimentally determined networks. Although the resemblance is not surprising (as it was built from experimentally determined reference interactomes), this confirms that *Z. mays* has the protein orthologs to generate a predicted interactome of normal topology. This lends confidence that the prediction process has not introduced systematic errors that altered the overall structure of the interactome.

### Co-expression of interacting proteins

The distribution of gene co-expression for interacting proteins in PiZeaM was different from what was reported in other predicted interactomes, including those of *A. thaliana* and *O. sativa*. When the entire distribution of correlation coefficients for every interacting protein pair was analyzed, it was found to be uniform and only slightly skewed to the right with the predicted *Z. mays* interologs, indicating a slightly higher likelihood of co-expression for interacting proteins (Figure 1C). This was not like the tight correlation shown in other plant interactomes (Geisler-Lee et al., 2007; Ho et al., 2012). Randomized interologs (random pairs of proteins from the interactome) displayed a left shifted higher peak in protein interactions distribution than the predicted interologs. A paired *T*-test was performed to compare correlations and a significant difference (*P*-value of 9.5 × 10^−39^) was found between random and predicted interologs. This showed that, though the difference was slight, there was a significant difference between the randomized network and PiZeaM.

### Go-enrichment for PiZeaM

When PiZeam was evaluated for enriched and depleted GO terms there was an overall enrichment of interactions of proteins localized to the chloroplast, mitochondrion, plasma membrane, nucleolus, and cytoplasm (Supplemental Table 3). Proteins localized to the chloroplast (GO: 9507), for example, occurred in 538 interactions in the network (*p*-value of 1.0 × 10^−128^ vs. occurrence by chance). Several biological processes were enriched in the entire interactome, including cell division and vitamin biosynthetic pathways (GO: 9110). There was also enrichment of processes thought to be plant specific, such as 14 proteins that have been predicted to be involved in defense against fungal pathogens (GO: 50382). PiZeaM is thus biased (enriched or depleted) for specific cellular and biological processes.

### Conserved subnetworks

**Figure 2:**
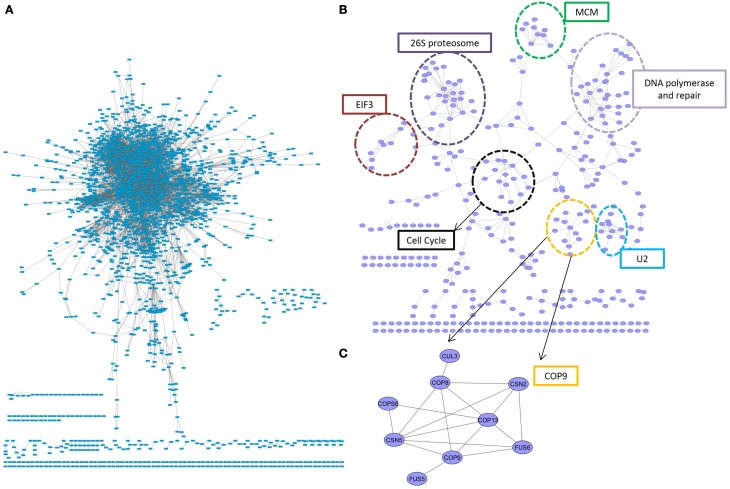
**Conserved subnetworks. (A)** A sub-network consisting of interactions identified by matching interologs in at least 4 (out of 13) reference species. The majority of proteins were found in a single connected network, with several smaller unconnected subnetworks. **(B)** A more stringent subnetwork of interactions with at least 5 interolog reference species. Clear clusters were concentrated in an organic layout by having more inter-group connections than between-group connections. These clusters consisted primarily of proteins with the same biological role, indicated by dotted circles. One such cluster **(C)** was an unconnected subnetwork consisting entirely of members of the COP9 signalosome. EIF3, elongation initation factor complex 3; U2, U2 splicosome; MCM, mini-chromosome maintenance proteins complex.

The most conserved interactions were identified in an initial analysis of the predicted interactome and its underlying biology. Highly conserved interactions represent ancient pathways that formed in early common ancestors and have remained intact as eukaryotes diverged into their extant forms. There was a large number of unique interactions (6650) with orthologs in more than 4 reference species (Figure 1A). These conserved interactions also had high confidence values in PiZeaM (1644 had CV greater than or equal to 100). This was not surprising as one of the factors used to determine the confidence level of a predicted interaction was the number of species (S) where such an interaction has been previously reported. Subnetworks were created for interactions with interologs in greater than 4 and greater than 5 species (Figures 2A,B). Overall, these included fewer proteins and a significantly smaller ratio of interactions per protein (3.0, 1.2, respectively, in Figures 2A,B) in comparison with the complete interactome (8.2). In the most stringently conserved subnet (5 species, Figure 2B), many well-known complexes such as the COP9 signalosome, the U2 splicosome, the mitotic spindle, histone interactions, and 26S proteosome are represented as interaction clusters (example in Figure 2C). The conserved subnetworks also showed connectivity between some clusters; i.e., the connections between the 26S proteosome and the translation initiation complex (Figure 2B upper left).

### Hypothesis generation and data mining in a disease resistance subnetwork

PiZeaM is like a detailed road and town map of an entire continent when visualized graphically. It is visually cluttered due to the number of nodes and edges (for example Figure 2A). However, it is useful when a small portion of interest is focused on (i.e., Figure 2C) for hypothesis generation and data mining. Identification of protein-protein interacting subnetworks in a given biological process in *Z. mays* is vital to achieve a better understanding of that process and how it connects to other processes.

### Data mining in biotic stress signaling

A map of the interconnections within the biotic stress signaling and response pathways was developed using a bait-and-prey approach. This approach was taken specifically to examine defense pathways against plant pathogens. All 154 proteins with the GO annotation “Response to other organism” were used as “bait” (see Disease subnet 1 in Supplemental File 1). These proteins include many known pathogen response proteins, such as *NON-EXPRESSION OF PATHOGEN RESISTANCE GENE 1* (NPR1), GRMZM2G076450 in *Z. mays* (Chern et al., 2001; Mou et al., 2003; Spoel et al., 2003), which is the key signaling protein for plant systemic acquired resistance (SAR) (Chern et al., 2001; Ferrari et al., 2003; Mou et al., 2003; Dong et al., 2006; Griebel and Zeier, 2008). The entire interactome was searched for proteins directly interacting (i.e., first neighbors) with the bait. This recovered 1424 “prey” proteins. Subsequent GO term enrichment analysis of the prey indicated enrichment for cytoplasm localized proteins, pyrophosphatases and acid anhydride hydrolases and proteins involved in ketone, small molecule, and primary metabolism (full list in Supplemental Table 4).

**Figure 3:**
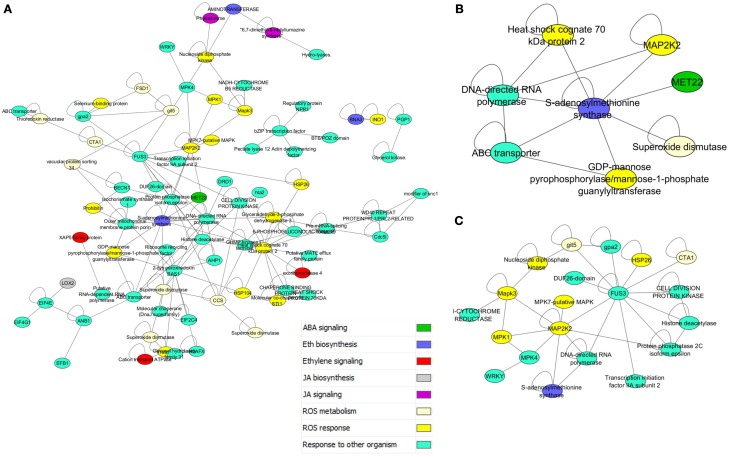
**Disease response and hormone subnetworks. (A)** Interaction between all proteins in PiZeaM with the gene ontology annotation “response to other organism,” which includes many disease response proteins, and their first neighbors from ROS and stress hormone signaling and metabolic pathways. **(B)** A nested subnetwork taken from **(A)** of MAPK signaling proteins and first neighbors. **(C)** A nested subnetwork taken from **(A)** of S-adenosyl methionine synthase (ethylene biosynthesis) and first neighbors.

The prey dataset was then limited to key regulators and metabolic proteins for reactive oxygen species (ROS), and the hormones jasmonic acid (JA), salicylic acid (SA), ethylene (ET), and abscisic acid (ABA) pathways (Ferrari et al., 2003; Baxter et al., 2014). This list of 81 proteins included 163 interactions, and formed a large connected subnetwork (Figure 3A). There were, however, no clear clusters of specific roles, unlike the highly conserved subnetworks in Figure 2B. The degree of cross connection is somewhat expected given that proteins such as enhanced disease susceptibility 1 protein (EDS1) have been shown to be involved in multiple signaling pathways, including the salicylic acid pathway and the regulation of the jasmonate pathway, to allow specific responses to pathogens (Heidrich et al., 2011). Regulators such as the EDS1-PAD4 protein (phytoalexin deficient 4) are involved in the response to the hemibiotrophic pathogen *Blumeria graminis*, the causal agent of powdery mildew on grasses (Parker et al., 1996; Yun et al., 2003; Wiermer et al., 2005). The subnetwork demonstrates that the three hormone pathways implicated in interactions with necrotrophic plant pathogens (Jasmonic acid, Abscisic acid, and Salicylic acid) (El-Zahaby et al., 1995; De Gara et al., 2003; Baxter et al., 2014) are physically interacting with proteins involved in ROS signaling. Interestingly, the influence of light flux rate and red/far-red shifts is connected to the network through GRMZM2G013478_P01, a predicted ROS response protein interacting with GRMZM2G092174_P01, a phytochrome protein (Griebel and Zeier, 2008; Moreno et al., 2009). Interactions such as these are presumed to be key to plant-microbe defense responses due to the interplay between light and susceptibility to pathogens (Mühlenbock et al., 2008; Roden and Ingle, 2009).

A nested subnetwork was also built using S-adenosyl methionine synthase (SAM) and its first neighbors (Figure 3B). SAM is a key step in the biosynthesis of the hormone ethylene, involved in responses to both biotic and abiotic stresses (Yang and Hoffman, 1984). This subnetwork included connections to a superoxide dismutase (which breaks down the ROS superoxide into another ROS hydrogen peroxide), and a ROS responsive GDP mannose pyrophosphorylase, an enzyme that generates an activated mannose important in n-linked glycosylation. SAM synthase is also predicted to interact with an ABC transporter and an RNA polymerase linked to biotic responses, and a MAPK signaling protein. Several additional mitogen activated protein kinases (MAPKs) were also included in the “response to other organism subnetwork” (Figure 3A), when these were isolated along with their interacting partners (Figure 3C). This illustrates connections between different layers of the signaling cascade. A number of other proteins were connected to this cascade, including a protein kinase involved in cell division, a WRKY transcription factor, an S-adenosyl methionine (SAM) synthase, and an uncharacterized protein with a domain of unknown function (DUF26).

## Discussion

### How accurate are interolog predictions in plant species?

The interolog method is useful in identifying interactions that are conserved between different organisms. This has been demonstrated in *A. thaliana* and other key organisms. It allows for a cost effective high-throughput analysis of potential protein-protein interactions, building on previous experimental evidence acquired from other species (De Bodt et al., 2009). It makes use of the Inparanoid method which has been shown to be able to compete with tree based methods for detection of orthology (Altenhoff and Dessimoz, 2009). Moreover, relying solely on experimental approaches to identify PPIs is technically challenging, resource intensive and time consuming, especially since relying on just one experimental method to identify PPIs can lead to false positives. Due to these limitations, the interolog method allows for novel hypotheses to be developed, using existing hard to obtain experimental data, including PPIs data gathered in other organisms (De Bodt et al., 2009).

After the predicted interactome for *A. thaliana* was released in 2007, a high throughput experimental interactome was created, making the comparison between the predicted interactome and the experimental intearctome possible. The two interactomes were compared to confirm that plant protein interactions can indeed be predicted using non-plant reference species. We compared the 72,266 interolog interactions in *A. thaliana* predicted by Geisler-Lee to the dataset of 37,645 experimentally determined PPIs collected at the BioArrayResource (http://bar.utoronto.ca/welcome.htm) (Toufighi et al., 2005). The observed overlap between the predicted and experimental datasets was 1450 interactions, compared to an expected overlap by chance of about 91 interactions (as two random subsets of (V^2^ + V)/2, where V is the number of proteins common to both sets). The observed overlap was calculated to be enriched 15-fold over chance, with a *p*-value of less than 10^−100^ in a simple 1° of freedom chi-square test. There is also considerable bias in experimentally determined interactions toward plant specific interactions, while the *A. thaliana* interolog predictions are focused on evolutionarily conserved interactions among eukaryotes identified by the ortholog methodology. Neither dataset is likely to be a complete map of all protein interactions in *A. thaliana*. The interactome of *S. cerevisiae* has over 230,000 known unique interactions for a very small genome (6600 genes). The number of interactions in a multicellular organism with over 30,000 proteins can be expected to be much higher with well over one million interactions, by simple extrapolation of genome size. Finding 1450 PPIs in the *A. thaliana* predicted interactome that were also experimentally validated is an indicator that the interolog method is reliable for the discovery of protein interactions in plants, even when using non-plant organisms as reference. All confidence levels of interactions performed much better than chance, with high confidence interactions (CV > 10) performing slightly better than low and medium confidence interactions. There was, however, no significant improvement in overlap with experimentally validated interactions (data not shown).

An important feature of PiZeaM is the inclusion of other “green” species such as *A. thaliana*, *Synechocystis*, and *O. sativa* as experimentally determined reference interactomes. This helps capture the unique aspects of plant pathways such as photosynthesis, cell wall formation, plant development, and disease resistance pathways. Earlier predicted interactomes, such as those of *A. thaliana* and *O. sativa* (Gu et al., 2011; Ho et al., 2012), had a significant number of interactions, but no plant references. The GO-term enrichment analysis in PiZeaM shows significant enrichment of chloroplast localized proteins and plant specific pathways, such as phytochrome light sensing, among the interactors. The successful execution of the methodology was in part measured by the examination of recaptured known conserved networks, including the 26s proteosome, ribosome subunits, DNA repair, splicosome, and COP9 signalosome interactions. The major differences in applying this method to *Z. mays* were the much larger genome and proteome of this species, which had undergone more extensive gene duplication than *A. thaliana* or *O. sativa*. Unlike model organisms, a high number of recent paralogs (inparalogs) in *Z. mays* are due to its polyploid genome, thus making most of these homeologs, or genes duplicated due to polyploidy (Adams and Wendel, 2005).

Although co-expression of gene transcripts is not necessarily required for a physical interaction between proteins, it is an observed trend reported in experimental interactomes that proteins that interact tend to be co-expressed. This is likely the effect of natural selection pressure on improving biological efficiency, i.e., not making proteins without all their necessary interacting partners present (Bhardwaj and Lu, 2005). The low correlation of interaction to co-expression in *Z. mays* seems to depart from these general observations and could possibly be due to the large number of recent paralogs that *Z. mays* has in its genome. Duplicated genes may have a faster divergence of RNA expression due to the relaxation of selection pressure, allowing mutation of promoter elements as well as coding sequences. Further analysis of other species with a similar genetic structure, such as wheat, might confirm or refute this hypothesis.

### Usefulness and novelty of PiZeaM

The interactome can be visualized as a field of circles (nodes) that represent proteins and connections (edges) between nodes that represent protein-protein interactions. Each node can be rated based on the number of connections, or the connectivity of that node. As stated previously, protein interactomes typically contain a few highly connected hubs, numerous smaller hubs, pipes, and free ends. This distribution is similar to that of other small-world network structures (Watts and Strogatz, 1998; De Silva et al., 2006; Gu et al., 2011). The small-world topology is a compromise between efficiency and robustness. Having fewer interacting partners involved in a pathway results in increased efficiency in terms of how fast a product or outcome can be processed. The highly connected hubs represent proteins that are conserved through different organisms and are under less selection pressure for mutations (Batada et al., 2006; Zotenko et al., 2008; Ning et al., 2010). For example, heat shock proteins (i.e., Hsp70, Hsp90) are vital for proper folding, assembly, and translocation and have been implicated in abiotic and biotic stress studies (Wang et al., 2004). As previously stated, these proteins are some of the highly connected hubs in the PiZeaM. A robust system includes redundant pathways, autoregulation, and feedback for increased stability. Understanding the interplay between efficiency and robustness is an emerging topic of interactomics that sheds light on the organization of various interactions that take place in organisms. This has led to recent advances in the mathematical field of graph theory to analyze and solve these real world problems. Moreover, there has been an increase in studies to determine the relationship between simple sub-networks and complete publicly available networks (Barabasi and Oltvai, 2004). An example would be studies aimed at comparing networks in maps and Internet social sites and drawing parallels from those studies to analyze biological networks. Another feature of small-world graphs is the enrichment of short path lengths reflecting the number of steps in a path between any two nodes (Supplemental Figure 1). This too was noted in predicted and experimentally determined interactomes such as the PRIN *O. sativa* interactome (Gu et al., 2011).

By mapping *Z. mays* proteins to orthologs in other species, prediction of function is also improved, which allowed superior annotation for *Z. mays* proteins in comparison to previously used methods (Geisler-Lee et al., 2007). Not just relying on orthology, the Geisler-Lee method used to build PiZeaM also utilizes interologs (interacting orthologs), co-expression, graph theory and gene ontology as additional layers of annotation of the network (Bhardwaj and Lu, 2005; Brown and Jurisica, 2005; Peterson et al., 2009). This is vital because orthologs across species are not always phylogenetically closer than paralogs (Koski and Golding, 2001). However, the interolog method makes the assumption that if an interaction occurs in the last common ancestor, and both proteins are retained after divergence, the interaction of the proteins is also retained after divergence.

In conclusion, PiZeaM represents a step forward in developing tools to utilize and integrate publically available genomic and proteomic data to improve our understanding of networks underlying plant-microbe interactions, breeding and development in *Z. mays*. Future work will analyze dynamic relationships in networks to determine causal relationships underlying *Z. mays* protein interactions.

### Conflict of interest statement

The authors declare that the research was conducted in the absence of any commercial or financial relationships that could be construed as a potential conflict of interest.

## Supplementary material

Supplemental File 1 **Cytoscape visualized interactome**. This file contains the maize predicted interactome as a Cytoscape ver 3.11 format file. Interactions shown are indicated and filterable by orthology (many-to-many and one-to-one), confidence value (numerical), and connectivity. The bait proteins used in the construction of subnetworks are indicated by pathway and gene name columns. Subnetworks shown in Figures 2, 3 are included, and colors of bait proteins correspond to that portrayed in the figures.

Supplemental Figure 1 **Shortest path length distribution**. This shows the frequency distribution shortest path length between every pairwise combination of proteins of the maize interactome using the NetworkAnalyzer tool provided by Cytoscape (ver. 3.11) plugins. A majority of the interactions in the maize interactome have a shortest path length between two and six.

Supplemental Table S1 **The predicted maize interactome**. The Master Interactome sheet contains all interolog predictions for the maize interactome using either many to many or one to one orthology. This table also lists the reference interactome including organism, orthologous proteins, type of experiment, publication identifier, and type of orthology used. Sheets containing excel versions of tables included in the manuscript are also provided for easy use in calculations. Other sheets contain unique interacting pairs for either one-to-one orthology or all unique interaction datasets. Analyses include a calculated confidence value (CV sheet) for each unique interaction, the number of times each interaction occurred in the reference data, and whether the interaction was a self-interaction or heterologous. A Node Property sheet provides annotation and shows the number of interactions each proteins has in either the unique or combined datasets. The Unique Edges sheet is a unique (each interaction represented once) combination of one to one and many to many interactions used in preparing the cytoscape visualization in Supplemental File 1.

Supplemental Table S2 **Microarray Accessions**. A listing of GEO accession numbers for all the Affymetrix microarrays downloaded to construct the co-expression matrix used in Figure 1C.

Supplemental Table S3 **GO enrichment analysis for PiZeaM**. The gene ontology terms associated with all 6004 proteins of PiZeaM were compared to the whole maize genome and ranked by multiple-hypothesis adjusted *P*-value (FDR). The analysis was done using the agriGO tool (http://bioinfo.cau.edu.cn/agriGO/) and Bingo Cytoscape Plugin.

Supplemental Table S4 **GO enrichment analysis for first neighbors of disease subnet 1**. PiZeaM had 1424 predicted interacting proteins for the 154 proteins with the gene ontology annotation “responds to other organism” (GO:0051707). These were compared to all maize proteins using the agriGO tool (http://bioinfo.cau.edu.cn/agriGO/).

## References

[B1] Adams K. L. Wendel J. F. (2005). Polyploidy and genome evolution in plants. Curr. Opin. Plant Biol. 8, 135–141. 10.1016/j.pbi.2005.01.001 15752992

[B2] Alon U. (2007). An Introduction to Systems Biology: Design Principles of Biological Circuits. Boca Raton, FL: Chapman & Hall/CRC.

[B3] Altenhoff A. M. Dessimoz C. (2009). Phylogenetic and functional assessment of orthologs inference projects and methods. PLoS Comput. Biol. 5:e1000262. 10.1371/journal.pcbi.1000262 19148271 PMC2612752

[B4] Aranda B. Achuthan P. Alam-Faruque Y. Armean I. Bridge A. Derow C.. (2010). The IntAct molecular interaction database in 2010. Nucleic Acids Res. 38, D525–D531. 10.1093/nar/gkp878 19850723 PMC2808934

[B5] Barabasi A.-L. Oltvai Z. N. (2004). Network biology: understanding the cell's functional organization. Nat. Rev. Genet. 5, 101–113. 10.1038/nrg1272 14735121

[B6] Batada N. Hurst L. Tyers M. (2006). Evolutionary and physiological importance of hub proteins. PLoS Comput. Biol. 2:e88. 10.1371/journal.pcbi.0020088 16839197 PMC1500817

[B7] Baxter A. Mittler R. Suzuki N. (2014). ROS as key players in plant stress signalling. J. Exp. Bot. 65, 1229–1240. 10.1093/jxb/ert375 24253197

[B8] Bhardwaj N. Lu H. (2005). Correlation between gene expression profiles and protein-protein interactions within and across genomes. Bioinformatics 21, 2730–2738. 10.1093/bioinformatics/bti398 15797912

[B9] Bork P. Jensen L. J. Von Mering C. Ramani A. K. Lee I. Marcotte E. M. (2004). Protein interaction networks from yeast to human. Curr. Opin. Struct. Biol. 14, 292–299. 10.1016/j.sbi.2004.05.003 15193308

[B10] Brown K. R. Jurisica I. (2005). Online predicted human interaction database. Bioinformatics 21, 2076–2082. 10.1093/bioinformatics/bti273 15657099

[B11] Carbon S. Ireland A. Mungall C. J. Shu S. Marshall B. Lewis S. (2009). AmiGO: online access to ontology and annotation data. Bioinformatics 25, 288–289. 10.1093/bioinformatics/btn615 19033274 PMC2639003

[B12] Chandler V. L. Brendel V. (2002). The maize genome sequencing project. Plant Physiol. 130, 1594–1597. 10.1104/pp.015594 12481042 PMC1540264

[B13] Chatr-Aryamontri A. Ceol A. Palazzi L. M. Nardelli G. Schneider M. V. Castagnoli L.. (2007). MINT: the molecular INTeraction database. Nucleic Acids Res. 35, D572–D574. 10.1093/nar/gkl950 17135203 PMC1751541

[B14] Chen J. Lalonde S. Obrdlik P. Noorani Vatani A. Parsa S. A. Vilariño C.. (2012). Uncovering Arabidopsis membrane protein interactome enriched in transporters using mating-based split ubiquitin assays and classification models. Front. Plant Sci. 3:124. 10.3389/fpls.2012.00124 22737156 PMC3380418

[B15] Chern M.-S. Fitzgerald H. A. Yadav R. C. Canlas P. E. Dong X. Ronald P. C. (2001). Evidence for a disease-resistance pathway in rice similar to the NPR1-mediated signaling pathway in Arabidopsis. Plant J. 27, 101–113. 10.1046/j.1365-313x.2001.01070.x 11489188

[B16] Cline M. S. Smoot M. Cerami E. Kuchinsky A. Landys N. Workman C.. (2007). Integration of biological networks and gene expression data using Cytoscape. Nat. Protoc. 2, 2366–2382. 10.1038/nprot.2007.324 17947979 PMC3685583

[B17] Consortium A. I. M. (2011). Evidence for network evolution in an Arabidopsis interactome map. Science 333, 601–607. 10.1126/science.1203877 21798944 PMC3170756

[B18] De Bodt S. Proost S. Vandepoele K. Rouzé P. Van De Peer Y. (2009). Predicting protein-protein interactions in Arabidopsis thaliana through integration of orthology, gene ontology and co-expression. BMC Genomics 10:288. 10.1186/1471-2164-10-288 19563678 PMC2719670

[B19] De Gara L. De Pinto M. C. Tommasi F. (2003). The antioxidant systems vis-à-vis reactive oxygen species during plant–pathogen interaction. Plant Physiol. Biochem. 41, 863–870. 10.1016/S0981-9428(03)00135-9

[B20] De Silva E. Thorne T. Ingram P. Agrafioti I. Swire J. Wiuf C.. (2006). The effects of incomplete protein interaction data on structural and evolutionary inferences. BMC Biol. 4:39. 10.1186/1741-7007-4-39 17081312 PMC1665463

[B21] Ding Y.-D. Chang J.-W. Guo J. Chen D.-J. Li S. Xu Q.. (2014). Prediction and functional analysis of the sweet orange protein-protein interaction network. BMC Plant Biol. 14:213. 10.1186/s12870-014-0213-7 25091279 PMC4236729

[B22] Dong W. Nowara D. Schweizer P. (2006). Protein polyubiquitination plays a role in basal host resistance of barley. Plant Cell 18, 3321–3331. 10.1105/tpc.106.046326 17114351 PMC1693960

[B23] Edgar R. Domrachev M. Lash A. E. (2002). Gene expression omnibus: NCBI gene expression and hybridization array data repository. Nucleic Acids Res. 30, 207–210. 10.1093/nar/30.1.207 11752295 PMC99122

[B24] El-Zahaby H. Gullner G. Kiraly Z. (1995). Effects of powdery mildew infection of barley on the ascorbate-glutathione cycle and other antioxidants in different host-pathogen interactions. Phytopathology 85, 1225–1230. 10.1094/Phyto-85-1225

[B25] Evlampiev K. Isambert H. (2008). Conservation and topology of protein interaction networks under duplication-divergence evolution. Proc. Natl. Acad. Sci. U.S.A. 105, 9863–9868. 10.1073/pnas.0804119105 18632555 PMC2481380

[B25a] FAOstat. (2009). Agriculture Organization of the United Nations. Statistical Database. Available online at: faostat.fao.org

[B26] Ferrari S. Plotnikova J. M. De Lorenzo G. Ausubel F. M. (2003). Arabidopsis local resistance to *Botrytis cinerea* involves salicylic acid and camalexin and requires EDS4 and PAD2, but not SID2, EDS5 or PAD4. Plant J. 35, 193–205. 10.1046/j.1365-313X.2003.01794.x 12848825

[B27] Flicek P. Amode M. R. Barrell D. Beal K. Brent S. Carvalho-Silva D.. (2012). Ensembl 2012. Nucleic Acids Res. 40, D84–D90. 10.1093/nar/gkr991 22086963 PMC3245178

[B28] Geer L. Y. Marchler-Bauer A. Geer R. C. Han L. He J. He S.. (2010). The NCBI BioSystems database. Nucleic Acids Res. 38, D492–D496. 10.1093/nar/gkp858 19854944 PMC2808896

[B29] Geisler-Lee J. O'toole N. Ammar R. Provart N. J. Millar A. H. Geisler M. (2007). A predicted interactome for Arabidopsis. Plant Physiol. 145, 317–329. 10.1104/pp.107.103465 17675552 PMC2048726

[B30] Geisler M. Fitzek E. (2011). A Predicted Interactome for Coffee (Coffea canephora var robusta). J. Plant Mol. Biol. Biotechnol. 2, 34–46.

[B31] Giot L. Bader J. S. Brouwer C. Chaudhuri A. Kuang B. Li Y.. (2003). A protein interaction map of *Drosophila melanogaster*. Science 302, 1727–1736. 10.1126/science.1090289 14605208

[B32] Gopinath R. K. You S.-T. Chien K.-Y. Swamy K. B. Yu J.-S. Schuyler S. C.. (2014). The Hsp90-dependent proteome is conserved and enriched for hub proteins with high levels of protein–protein connectivity. Genome Biol. Evol. 6, 2851–2865. 10.1093/gbe/evu226 25316598 PMC4224352

[B33] Griebel T. Zeier J. (2008). Light regulation and daytime dependency of inducible plant defenses in Arabidopsis: phytochrome signaling controls systemic acquired resistance rather than local defense. Plant Physiol. 147, 790–801. 10.1104/pp.108.119503 18434604 PMC2409012

[B34] Gu H. Zhu P. Jiao Y. Meng Y. Chen M. (2011). PRIN: a predicted rice interactome network. BMC Bioinformatics 12:161. 10.1186/1471-2105-12-161 21575196 PMC3118165

[B35] Guan Y. Myers C. L. Lu R. Lemischka I. R. Bult C. J. Troyanskaya O. G. (2008). A genomewide functional network for the laboratory mouse. PLoS Comput. Biol. 4:e1000165. 10.1371/journal.pcbi.1000165 18818725 PMC2527685

[B36a] Guo J. Wang J. Xi L. Huang W.-D. Liang J. Chen J.-G. (2009). RACK1 is a negative regulator of ABA responses in *Arabidopsis*. J. Exp. Bot. 60, 3819–3833. 10.1093/jxb/erp221 19584117 PMC2736894

[B36] Gursoy A. Keskin O. Nussinov R. (2008). Topological properties of protein interaction networks from a structural perspective. Biochem. Soc. Trans. 36, 1398–1403. 10.1042/BST0361398 19021563 PMC7243876

[B37] Heidrich K. Wirthmueller L. Tasset C. Pouzet C. Deslandes L. Parker J. E. (2011). Arabidopsis EDS1 connects pathogen effector recognition to cell compartment–specific immune responses. Science 334, 1401–1404. 10.1126/science.1211641 22158818

[B38] Hershko A. Ciechanover A. (1998). The ubiquitin system. Annu. Rev. Biochem. 67, 425–479. 10.1146/annurev.biochem.67.1.425 9759494

[B39] Ho C.-L. Wu Y. Shen H.-B. Provart N. Geisler M. (2012). A predicted protein interactome for rice. Rice 5:15. 10.1186/1939-8433-5-15 24279740 PMC4883691

[B40] Kaiser P. Huang L. (2005). Global approaches to understanding ubiquitination. Genome Biol. 6:233. 10.1186/gb-2005-6-10-233 16207362 PMC1257457

[B41] Kim W.-Y. Kang S. Kim B.-C. Oh J. Cho S. Bhak J.. (2008). SynechoNET: integrated protein-protein interaction database of a model cyanobacterium Synechocystis sp. PCC 6803. BMC Bioinformatics 9:S20. 10.1186/1471-2105-9-S1-S20 18315852 PMC2259421

[B42] Koonin E. V. (2005). Orthologs, paralogs, and evolutionary genomics 1. Annu. Rev. Genet. 39, 309–338. 10.1146/annurev.genet.39.073003.114725 16285863

[B43] Koski L. B. Golding G. B. (2001). The closest BLAST hit is often not the nearest neighbor. J. Mol. Evol. 52, 540–542. 10.1007/s002390010184 11443357

[B44] Lalonde S. Sero A. Pratelli R. Pilot G. Chen J. Sardi M. I. (2010). A membrane protein/signaling protein interaction network for Arabidopsis version AMPv2. Front. Physiol. 1:24 10.3389/fphys.2010.00024 PMC3059934 21423366

[B45] Li L. Stoeckert C. J. Roos D. S. (2003). OrthoMCL: identification of ortholog groups for eukaryotic genomes. Genome Res. 13, 2178–2189. 10.1101/gr.1224503 12952885 PMC403725

[B46] Li S. Armstrong C. M. Bertin N. Ge H. Milstein S. Boxem M.. (2004). A map of the interactome network of the metazoan *C. elegans*. Science 303, 540–543. 10.1126/science.1091403 14704431 PMC1698949

[B47] Lightfoot D. A. (2014). The Soybean Genome Database (SoyGD): a genome, proteome and interactome viewer based on cultivar forrest, in Plant and Animal Genome XXII Conference: Plant and Animal Genome (San Diego, CA).

[B48] Liu L. White M. J. Macrae T. H. (1999). Transcription factors and their genes in higher plants. Eur. J. Biochem. 262, 247–257. 10.1046/j.1432-1327.1999.00349.x 10336605

[B49] Maere S. Heymans K. Kuiper M. (2005). BiNGO: a Cytoscape plugin to assess overrepresentation of gene ontology categories in biological networks. Bioinformatics 21 3448–3449. 10.1093/bioinformatics/bti551 15972284

[B50] Martinelli F. Reagan R. L. Uratsu S. L. Phu M. L. Albrecht U. Zhao W.. (2013). Gene regulatory networks elucidating Huanglongbing disease mechanisms. PLoS ONE 8:e74256. 10.1371/journal.pone.0074256 24086326 PMC3783430

[B51] Martinelli F. Uratsu S. L. Albrecht U. Reagan R. L. Phu M. L. Britton M.. (2012). Transcriptome profiling of citrus fruit response to huanglongbing disease. PLoS ONE 7:e38039. 10.1371/journal.pone.0038039 22675433 PMC3364978

[B52] Moreno J. E. Tao Y. Chory J. Ballaré C. L. (2009). Ecological modulation of plant defense via phytochrome control of jasmonate sensitivity. Proc. Natl. Acad. Sci. U.S.A. 106, 4935–4940. 10.1073/pnas.0900701106 19251652 PMC2660767

[B53] Mosca R. Pache R. A. Aloy P. (2012). The role of structural disorder in the rewiring of protein interactions through evolution. Mol. Cell. Proteomics 11:M111.014969. 10.1074/mcp.M111.014969 22389433 PMC3394948

[B54] Mou Z. Fan W. Dong X. (2003). Inducers of plant systemic acquired resistance regulate NPR1 function through redox changes. Cell 113, 935–944. 10.1016/S0092-8674(03)00429-X 12837250

[B55] Mühlenbock P. Szechyñska-Hebda M. Płaszczyca M. Baudo M. Mateo A. Mullineaux P. M.. (2008). Chloroplast signaling and LESION SIMULATING DISEASE1 regulate crosstalk between light acclimation and immunity in Arabidopsis. Plant Cell 20, 2339–2356. 10.1105/tpc.108.059618 18790826 PMC2570729

[B56] Narayanan M. Vetta A. Schadt E. E. Zhu J. (2010). Simultaneous clustering of multiple gene expression and physical interaction datasets. PLoS Comput. Biol. 6:e1000742. 10.1371/journal.pcbi.1000742 20419151 PMC2855327

[B57] Nejad E. S. Askari H. Soltani S. (2012). Regulatory TGACG-motif may elicit the secondary metabolite production through inhibition of active Cyclin-dependent kinase/Cyclin complex. Plant Omics 5, 553.

[B58] Ning K. Ng H. Srihari S. Leong H. Nesvizhskii A. (2010). Examination of the relationship between essential genes in PPI network and hub proteins in reverse nearest neighbor topology. BMC Bioinformatics 11:505. 10.1186/1471-2105-11-505 20939873 PMC3098085

[B59] Ohbayashi T. Makino Y. Tamura T.-A. (1999). Identification of a mouse TBP-like protein (TLP) distantly related to the Drosophila TBP-related factor. Nucleic Acids Res. 27, 750–755. 10.1093/nar/27.3.750 9889269 PMC148243

[B60] Ostlund G. Schmitt T. Forslund K. Kastler T. Messina D. N. Roopra S.. (2010). InParanoid 7: new algorithms and tools for eukaryotic orthology analysis. Nucleic Acids Res. 38, D196–D203. 10.1093/nar/gkp931 19892828 PMC2808972

[B61] Parker J. E. Holub E. B. Frost L. N. Falk A. Gunn N. D. Daniels M. J. (1996). Characterization of eds1, a mutation in Arabidopsis suppressing resistance to *Peronospora parasitica* specified by several different RPP genes. Plant Cell 8, 2033–2046. 10.1105/tpc.8.11.2033 8953768 PMC161332

[B62] Peterson M. E. Chen F. Saven J. G. Roos D. S. Babbitt P. C. Sali A. (2009). Evolutionary constraints on structural similarity in orthologs and paralogs. Protein Sci. 18, 1306–1315. 10.1002/pro.143 19472362 PMC2774440

[B63] Ranum P. Peña-Rosas J. P. Garcia-Casal M. N. (2014). Global maize production, utilization, and consumption. Ann. N.Y. Acad. Sci. 1312, 105–112. 10.1111/nyas.12396 24650320

[B64] Renewable Fuels Association. (2012). Accelerating Industry Innovation: 2012 Ethanol Industry Outlook. Available online at: www.ethanolrfa.org

[B65] Roden L. C. Ingle R. A. (2009). Lights, rhythms, infection: the role of light and the circadian clock in determining the outcome of plant–pathogen interactions. Plant Cell 21, 2546–2552. 10.1105/tpc.109.069922 19789275 PMC2768925

[B66] Rual J.-F. Venkatesan K. Hao T. Hirozane-Kishikawa T. Dricot A. Li N.. (2005). Towards a proteome-scale map of the human protein-protein interaction network. Nature 437, 1173–1178. 10.1038/nature04209 16189514

[B67] Rudrabhatla P. Reddy M. Rajasekharan R. (2006). Genome-wide analysis and experimentation of plant serine/ threonine/tyrosine-specific protein kinases. Plant Mol. Biol. 60, 293–319. 10.1007/s11103-005-4109-7 16429265

[B68] Salwinski L. Miller C. S. Smith A. J. Pettit F. K. Bowie J. U. Eisenberg D. (2004). The database of interacting proteins: 2004 update. Nucleic Acids Res. 32, D449–D451. 10.1093/nar/gkh086 14681454 PMC308820

[B69] Schnable P. S. Ware D. Fulton R. S. Stein J. C. Wei F. Pasternak S.. (2009). The B73 maize genome: complexity, diversity, and dynamics. Science 326, 1112–1115. 10.1126/science.1178534 19965430

[B70] Schuette S. Piatkowski B. Corley A. Lang D. Geisler M. (2015). Predicted protein-protein interactions in the moss *Physcomitrella patens*: a new bioinformatic resource. BMC Bioinformatics 16:89. 10.1186/s12859-015-0524-1 25885037 PMC4384322

[B71] Shannon P. Markiel A. Ozier O. Baliga N. S. Wang J. T. Ramage D.. (2003). Cytoscape: a software environment for integrated models of biomolecular interaction networks. Genome Res. 13, 2498–2504. 10.1101/gr.1239303 14597658 PMC403769

[B72] Soltes G. Muller E. D'aversa T. G. (2011). Ubiquitin, ubiquitination, and proteasomal degradation in the eukaryotic cell: a review. Bios 82, 64–71. 10.1893/011.082.0303

[B73] Spoel S. H. Koornneef A. Claessens S. M. Korzelius J. P. Van Pelt J. A. Mueller M. J.. (2003). NPR1 modulates cross-talk between salicylate-and jasmonate-dependent defense pathways through a novel function in the cytosol. Plant Cell 15, 760–770. 10.1105/tpc.009159 12615947 PMC150028

[B74] Stark C. Breitkreutz B.-J. Reguly T. Boucher L. Breitkreutz A. Tyers M. (2006). BioGRID: a general repository for interaction datasets. Nucleic Acids Res. 34, D535–D539. 10.1093/nar/gkj109 16381927 PMC1347471

[B75] Stuart J. M. Segal E. Koller D. Kim S. K. (2003). A gene-coexpression network for global discovery of conserved genetic modules. Science 302, 249–255. 10.1126/science.1087447 12934013

[B76] Taylor I. W. Linding R. Warde-Farley D. Liu Y. Pesquita C. Faria D.. (2009). Dynamic modularity in protein interaction networks predicts breast cancer outcome. Nat. Biotechnol. 27, 199–204. 10.1038/nbt.1522 19182785

[B77] Toufighi K. Brady S. M. Austin R. Ly E. Provart N. J. (2005). The botany array resource: e-northerns, expression angling, and promoter analyses. Plant J. 43, 153–163. 10.1111/j.1365-313X.2005.02437.x 15960624

[B78] Wachi S. Yoneda K. Wu R. (2005). Interactome-transcriptome analysis reveals the high centrality of genes differentially expressed in lung cancer tissues. Bioinformatics 21, 4205–4208. 10.1093/bioinformatics/bti688 16188928 PMC4631381

[B79] Wang W. Vinocur B. Shoseyov O. Altman A. (2004). Role of plant heat-shock proteins and molecular chaperones in the abiotic stress response. Trends Plant Sci. 9, 244–252. 10.1016/j.tplants.2004.03.006 15130550

[B80] Watts D. J. Strogatz S. H. (1998). Collective dynamics of /‘small-world/’ networks. Nature 393, 440–442. 10.1038/30918 9623998

[B81] Wiermer M. Feys B. J. Parker J. E. (2005). Plant immunity: the EDS1 regulatory node. Curr. Opin. Plant Biol. 8, 383–389. 10.1016/j.pbi.2005.05.010 15939664

[B82] Yang S. F. Hoffman N. E. (1984). Ethylene biosynthesis and its regulation in higher plants. Annu. Rev. Plant Physiol. 35, 155–189. 10.1146/annurev.pp.35.060184.001103

[B83] Youens-Clark K. Buckler E. Casstevens T. Chen C. Declerck G. Derwent P.. (2011). Gramene database in 2010: updates and extensions. Nucleic Acids Res. 39 D1085–D1094. 10.1093/nar/gkq1148 21076153 PMC3013721

[B84] Yu H. Braun P. Yıldırım M. A. Lemmens I. Venkatesan K. Sahalie J.. (2008). High-quality binary protein interaction map of the yeast interactome network. Science 322, 104–110. 10.1126/science.1158684 18719252 PMC2746753

[B85] Yun B. W. Atkinson H. A. Gaborit C. Greenland A. Read N. D. Pallas J. A.. (2003). Loss of actin cytoskeletal function and EDS1 activity, in combination, severely compromises non−host resistance in Arabidopsis against wheat powdery mildew. Plant J. 34, 768–777. 10.1046/j.1365-313X.2003.01773.x 12795697

[B86] Zotenko E. Mestre J. O'leary D. Przytycka T. (2008). Why do hubs in the yeast protein interaction network tend to be essential: reexamining the connection between the network topology and essentiality. PLoS Comput. Biol. 4:e1000140. 10.1371/journal.pcbi.1000140 18670624 PMC2467474

