# Supplementary material for: A predicted protein interactome identifies conserved global networks and disease resistance subnetworks in maize
Source: Front Genet. 2015 Jun 4;6:201. doi: 10.3389/fgene.2015.00201 (PMC4454876; doi:10.3389/fgene.2015.00201)
Supplement: Supplemental Figure 1 — Shortest path length distribution. This shows the frequency distribution shortest path length between every pairwise combination of proteins of the maize interactome using the NetworkAnalyzer tool provided by Cytoscape (ver. 3.11) plugins. A majority of the interactions in the maize interactome have a shortest path length between two and six. [file Image1.PDF]

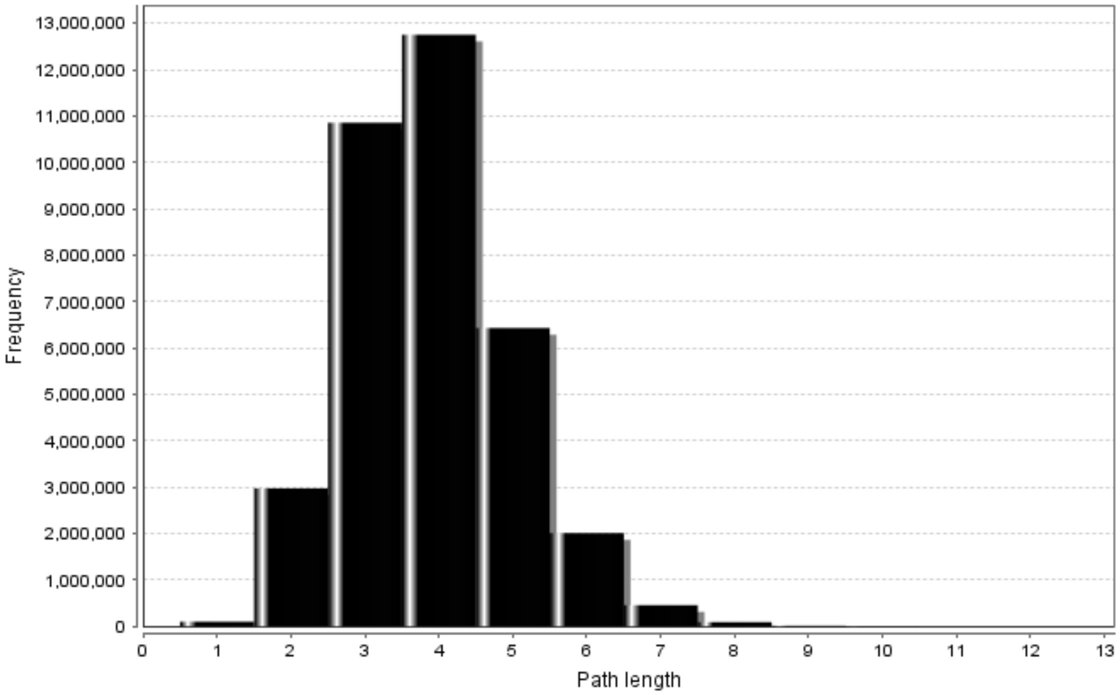

S**upplemental Figure 1. NetworkAnalzyer.** This **s**hows the path length frequency of the maize interactome using the NetworkAnalyzer tool provided by Cytoscape plugins. A majority of the interactions in the maize interactome have a path length between two and six.
